# Personalizezed Hemodynamic Optimization Using Stroke Volume, Pulse Pressure Variation, and Continuous Cardiac Index in Major Liver Surgery: A Randomized Controlled Trial

**DOI:** 10.3390/jpm15100457

**Published:** 2025-09-30

**Authors:** Francisco Javier Redondo Calvo, Víctor Baladrón González, David Padilla Valverde, Jorge Redondo Sánchez, Pedro Juan Villarejo Campos, Omar Montenegro Herrera, Patricia Faba Martín, Rubén Villazala González, Raquel Bodoque Villar, Juan Fernando Padin, José Ramón Muñoz-Rodríguez, Natalia Bejarano Ramírez

**Affiliations:** 1 Department of Anesthesiology and Critical Care Medicine, University General Hospital, 13004 Ciudad Real, Spain; vbaladron@sescam.jccm.es (V.B.G.); jredondo@sescam.jccm.es (J.R.S.); ommontenegro@sescam.jccm.es (O.M.H.); pfaba@sescam.jccm.es (P.F.M.); rvillazala@sescam.jccm.es (R.V.G.); 2 Faculty of Medicine, Universidad de Castilla-La Mancha, 13071 Ciudad Real, Spain; fernando.padin@uclm.es (J.F.P.); jmunozrodriguez@sescam.jccm.es (J.R.M.-R.); 3 Translational Research Group, GAI of Ciudad Real, Spain, Research Institute of Castilla-La Mancha (IDISCAM), 13004 Ciudad Real, Spain; rbodoquev@sescam.jccm.es (R.B.V.); nbejarano@sescam.jccm.es (N.B.R.); 4 Department of Surgery, Universitary General Hospital, 13004 Ciudad Real, Spain; 5 Department of Surgery, Universitary Hospital Fundación Jiménez Díaz, 28040 Madrid, Spain; villarejocampos@yahoo.es; 6 Department of Pediatrics, University General Hospital, 13004 Ciudad Real, Spain

**Keywords:** resection hepatic, stroke volume variation, pulse pressure variation, goal-directed hemodynamic therapy, intraoperative bleeding

## Abstract

**Background/Objectives**: The aim of this study was to evaluate fluid administration and intraoperative bleeding of patients who had major hepatic resection. We used artery pulse contour analysis monitor (ProAQT™) and personalized hemodynamic target-guided therapy, in which the administration of fluid, inotropes and vasopressors is guided by stroke volume, pulse pressure variation (SVV, PPV) and continuous cardiac index (CI). **Methods**: This trial was a prospective, randomized, parallel-group in adults scheduled for major hepatic resection. Participants were randomly assigned in equal numbers to one of two groups: (1) a control group receiving conventional perioperative care, and (2) an intervention group managed with goal-directed hemodynamic therapy guided by radial artery pulse contour analysis. **Results**: 45 patients were randomized to the GDHT (n = 16) and control group (n = 19). Blood loss was significantly higher in the control group than in GDHT group (728.13 ± 618.59 versus 292.63 ± 274.06, *p* = 0.009). The number of patients receiving intraoperative transfusion was significantly higher in the first group (6 ± 16 versus 0 ± 19, *p* = 0.005). Total volume infused was significantly higher in control group (CG) than in GDHT group (GG) (2853.13 ± 1432.18 versus 1125.79 ± 751.2, *p* = 0.001). **Conclusions**: Personalized goal-directed therapy optimizes intraoperative fluid administration during major liver resection and reduces blood transfusion.

## 1. Introduction

Intraoperative hemorrhage remains one of the leading contributors to morbidity and mortality during liver surgery [1]. To mitigate bleeding, surgical strategies often focus on limiting hepatic blood flow. Among these, the Pringle maneuver (PM), which involves clamping the portal triad, is the most commonly employed technique. However, its clinical value has been debated due to potential risks such as hemodynamic instability and ischemia–reperfusion injury [2].

A widely adopted approach to minimize blood loss during hepatic resections is maintaining a low central venous pressure (CVP), typically below 5–6 cm H_2_O, through fluid restriction [3]. Once the resection is complete, fluid resuscitation is generally used to restore euvolemia.

Traditional cardiac filling pressures, including CVP and pulmonary artery occlusion pressure (PAOP), have proven unreliable in predicting fluid responsiveness [4]. CVP readings can be influenced by multiple intraoperative factors such as mechanical ventilation, PM application, changes in peripheral resistance, and elevated intra-abdominal pressure [5]. Moreover, some studies have reported no significant correlation between low CVP and reduced bleeding [6].

Currently, many surgical teams favor restrictive fluid management protocols during liver resections, opting not to rely on cardiac filling pressures for guidance [7].

Dynamic preload indicators such as stroke volume variation (SVV) and pulse pressure variation (PPV) are grounded in the physiological interplay between cardiac and pulmonary systems. These metrics are extracted from circulatory oscillations induced by shifts in intrathoracic pressure during volume-controlled mechanical ventilation. Evidence supports their utility in forecasting fluid responsiveness in patients under mechanical ventilation [8].

More recently, the ratio of PPV (pressure-derived) to SVV (flow-derived) has been introduced as a surrogate marker for dynamic arterial elastance (Eadyn). This parameter offers a means to distinguish between hypotension caused by arterial vasodilation and that due to hypovolemia. Eadyn may also serve as a predictor of vascular tone modulation following fluid administration, particularly in relation to norepinephrine requirements [9].

Personalized goal-directed hemodynamic therapy (GDHT) involves tailoring the administration of fluids, inotropes, and vasopressors based on continuous monitoring of cardiac output and other hemodynamic variables. Its primary objective is to minimize perioperative complications, potentially lowering both morbidity and mortality rates [10].

A substantial body of research has demonstrated that perioperative GDHT can enhance clinical outcomes, especially in patients undergoing abdominal procedures [11,12], as well as in trauma and orthopedic surgeries [13]. Moreover, GDHT protocols incorporating PPV have shown promising results in improving patient prognosis [14].

The aim of the current study of patients undergoing major hepatic resection was comparing standard perioperative (control Group) to hemodynamic management based on PPV, SVV, continuous CO trending and dynamic arterial elastance using radial artery pulse contour analysis (GDHT group). We hypothesize that the personalized use of this treatment regimen following liver resection leads to a reduction in the total volume infused and in intraoperative transfusion (primary endpoint) and reduced postoperative complications and length of hospital stay (secondary endpoint).

## 2. Materials and Methods

### 2.1. Study Design and Protocol

This prospective, randomized clinical trial was conducted at the Departments of Anesthesiology and Intensive Care Medicine and Surgery of the University General Hospital of Ciudad Real, Spain. The study received approval from the institutional ethics committee (protocol code RD223/04. Date of approval: 25 November 2014, Acta 1/2014) and was registered at ClinicalTrials.gov (NCT04517409). Patients scheduled for hepatic resection involving more than two liver segments were considered eligible. The primary indications for hepatic resection in our study were colorectal liver metastases and hepatocellular carcinoma. Exclusion criteria included: age under 18 years, body weight below 50 kg or above 150 kg, irregular cardiac rhythm, severe cardiovascular disease (e.g., chronic heart failure, valvular pathology, cardiomyopathy), and advanced liver dysfunction (Child-Pugh class B or C). Written informed consent was obtained from all participants prior to enrollment.

### 2.2. Randomization and Group Allocation

Eligible patients were randomly assigned in a 1:1 ratio to one of two groups: (1) standard perioperative care (Control group), or (2) goal-directed hemodynamic therapy (GDHT group) guided by radial artery pulse contour analysis. Randomization was performed the day before surgery by the lead investigator responsible for anesthesia, using sealed opaque envelopes stored in non-transparent containers. Only patients were blinded to group allocation; blinding of care providers and investigators was not feasible due to the use of cardiac index monitoring devices.

### 2.3. Intraoperative Management

All patients received balanced general anesthesia with intravenous induction and neuromuscular blockade, administered at the discretion of the attending anesthesiologist. Depth of anesthesia was monitored using bispectral index (BIS), with maintenance via sevoflurane targeting a BIS range of 40–60. Central venous catheterization and invasive radial arterial pressure monitoring were performed based on anesthesiologist preference.

Standard monitoring included five-lead electrocardiography, pulse oximetry, and non-invasive blood pressure measurement. At least one peripheral intravenous line was established. Intraoperative goals included maintaining oxygen saturation >94%, normothermia, and heart rate <100 beats per minute. Mechanical ventilation with an inspired oxygen fraction of 60% was used to maintain arterial carbon dioxide tension (PaCO_2_) between 4.7 and 6.0 kPa, with a positive end-expiratory pressure (PEEP) of 4–6 mmHg and tidal volumes of 6–8 mL/kg.

Ephedrine was administered in cases of hemodynamic instability (mean arterial pressure < 65 mmHg). If repeated boluses were ineffective, norepinephrine infusion was initiated. In the event of acute blood loss (>150 mL/min or >500 mL), colloid solutions (Gelaspan^®^ 40/mg) were administered within the recommended maximum dose (30 mL/kg).

In both groups, blood loss was compensated with colloid infusion at a 1:1 ratio. Packed red blood cells were transfused when hemoglobin levels fell below 10 g/dL in patients with cardiac comorbidities, or below 7 g/dL in those without.

Hemodynamic parameters were recorded every 15 min. Arterial and central venous blood gas analyses were performed at the beginning, midpoint, and end of hepatic resection. At the conclusion of surgery, total catecholamine usage, estimated blood loss, urine output, and fluid administration were documented. The time from surgery completion to extubation was also recorded.

### 2.4. GDHT Group

In the GDHT group, the arterial catheter was additionally connected to a cardiac index trending monitor (ProAQT™, PULSION Medical Systems SE, Munich, Germany).

**Figure 1:**
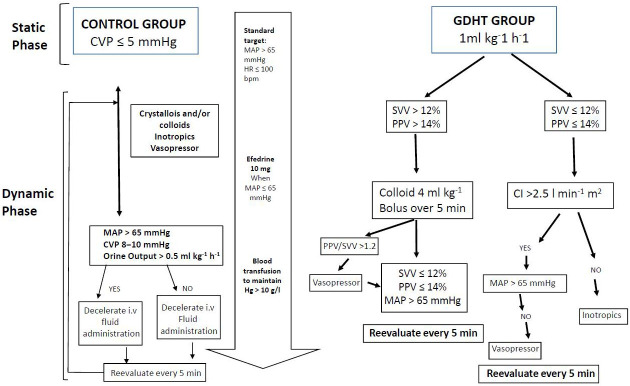
Intraoperative fluid management.

Fluid management followed a predefined protocol (Figure 1). During the static phase (prior to hepatic resection), all patients received a continuous infusion of balanced crystalloid solution (Ringer’s lactate) at a rate of 1 mL·kg^−1^·h^−1^.

Following hepatic resection, the dynamic phase commenced with an initial hemodynamic assessment based on pulse pressure variation (PPV), cardiac index (CI), and mean arterial pressure (MAP), as outlined in Figure 1. Preload optimization was achieved by administering 4 mL·kg^−1^ boluses of colloid solution every 5 min until PPV was <14% or stroke volume variation (SVV) was <12%. At this point, the patient’s individualized, preload-optimized CI was established and used as the target hemodynamic parameter for the remainder of the procedure.

If the optimized CI was below 2.5 L·min^−1^·m^−2^, inotropic support was initiated to reach this minimum threshold, serving as a safety measure to prevent low cardiac output. If PPV and CI were within target ranges but MAP remained <65 mmHg or the PPV/SVV ratio exceeded 1.2, vasopressor therapy was started.

After the initial assessment, patients were re-evaluated every 15 min throughout the intraoperative period to ensure maintenance of hemodynamic parameters in accordance with the study algorithm (Figure 1).

### 2.5. Control Group

During the static phase (prior to hepatic resection), patients in the control group received a continuous infusion of balanced crystalloid solution with the goal of maintaining a central venous pressure (CVP) of approximately 5 mmHg.

In the dynamic phase (after hepatic resection), fluid therapy—including colloid administration—as well as vasopressor and inotropic support were provided at the discretion of the attending anesthesiologist, based on CVP, MAP, and urine output. Cardiac output monitoring was not performed in this group. Intraoperative management goals were intentionally flexible to avoid extremes of clinical practice and to reflect real-world variability.

### 2.6. Postoperative Management

All patients were monitored in the post-anesthesia care unit (PACU) prior to transfer to the surgical ward. Arterial and central venous blood gas analyses were performed immediately before PACU discharge, and the duration of PACU stay was recorded in minutes.

Twenty-four hours after surgery, data were collected on catecholamine use, estimated blood loss, urine output, and total fluid administration. Postoperative complications and length of hospital stay were documented. Complications were predefined in the study protocol and categorized as follows: Infectious (respiratory, abdominal, urinary tract, or surgical site infections), Respiratory (mechanical ventilation > 24 h, failed extubation), Cardiovascular (atrial fibrillation, acute myocardial infarction, ventricular fibrillation, pulmonary edema, hypotension (MAP < 50 mmHg), stroke), Abdominal (reoperation, gastrointestinal bleeding, hepatic dysfunction), Renal (acute kidney injury), Other (wound dehiscence, deep vein thrombosis).

### 2.7. Endpoints

#### 2.7.1. Primary Endpoint

The primary endpoint was the volume of fluid administered intraoperatively and postoperatively, as well as the number of blood transfusions.

#### 2.7.2. Secondary Endpoints

Secondary endpoints included intraoperative laboratory parameters, use of vasopressors, duration of hepatic resection, and total surgical time. Postoperative complications were recorded based on patient chart review and direct ward visits by investigators. Definitions of complications were updated to align with current standards [15].

Additional outcomes included length of hospital stay (in days), duration of intensive care unit stay, and all-cause postoperative mortality. Baseline data collected included sociodemographic characteristics, ASA physical status classification [16], comorbidities, and preoperative hemoglobin levels.

### 2.8. Statistical Analysis

Quantitative variables are presented as mean ± standard error of the mean (SEM) and visualized using box plots. Group comparisons (Control vs. GDHT) were performed using Student’s *t*-test or the Mann–Whitney U test, depending on the normality of data distribution.

For within-group comparisons of hemodynamic changes in the GDHT group, paired Student’s *t*-tests or Wilcoxon signed-rank tests were used, based on normality assessment. A *p*-value < 0.05 was considered statistically significant, with a 95% confidence interval (CI). Statistical analyses were conducted using SPSS version 24.0 (IBM Corp., New York, NY, USA).

## 3. Results

**Figure 2:**
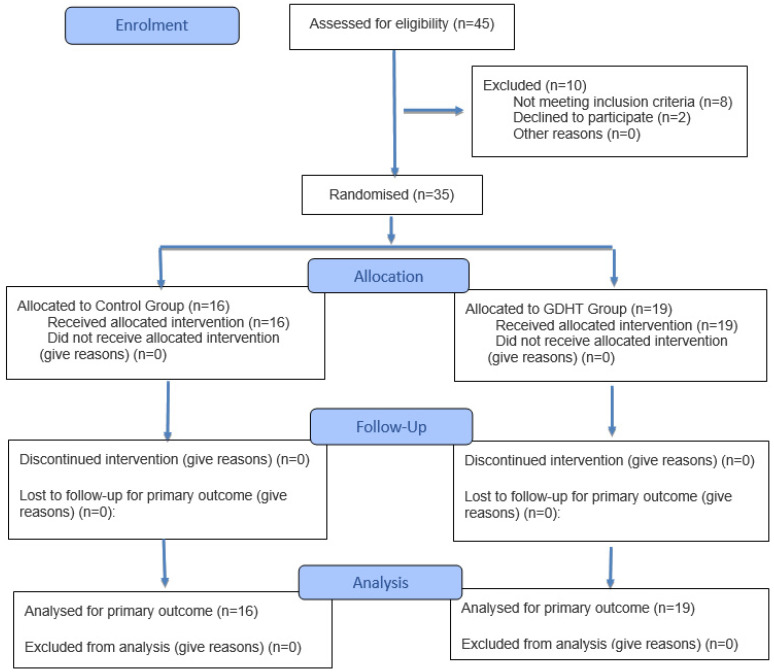
Flowchart of the patients in this study.

The study was performed for two years. In this period, 45 patients were scheduled for open large liver resection (two or more liver segments). No patient was excluded or dropped out of the study after randomization (N = 35). There were 16 patients in the control group and 19 patients in the GDHT group (Figure 2).

There were no significant differences between the groups regarding to demographics, baseline laboratory parameters and surgical characteristics (Table 1).

### 3.1. Intra- and Postoperative Laboratory Parameters

There was no significant difference in Laboratory parameters before, after resection hepatic and third postoperative day (Table 2).

### 3.2. Fluids and Catecholamines

#### 3.2.1. Fluid Management

Blood loss was significantly higher in the control group than in GDHT group (728.13 ± 618.59 versus 292.63 ± 274.06, *p* = 0.009). The number of patients receiving intraoperative transfusion was significantly higher in the first group (6 ± 16 versus 0 ± 19, *p* = 0.005). However, in the postoperative (Intensive Unit) transfusion there was no significant differences (Table 3).

Total volume infused was significantly higher in control group (CG) than in GDHT group (GG) (2853.13 ± 1432.18 versus 1125.79 ± 751.2, *p* = 0.001), although more marked in static phase (1403.13 ± 1146.51 versus 276.32 ± 189.56, *p* = 0.001). Also, urine output was significantly higher in control group than in GDHT group (430.63 ± 310.26 versus 206.84 ± 133.2, *p* = 0.007) (Table 3).

#### 3.2.2. Vasopressor

As listed in Table 3, the number of patients receiving intraoperative vasopressor was equal in the two groups (CG:6 ± 16 versus GG:6 ± 19, *p* = 0.495). Few patients required vasopressors postoperatively with no significant difference between the two groups (CG:6 ± 16 versus GG:4 ± 19, *p* = 0.311)

### 3.3. Complications and Outcome

The overall number of complications was similar between the two groups (CG: 8 ± 16 versus GG: 7 ± 18, *p* = 0.38) with no significant difference. Further, there were no significant differences in the duration of stay in the intensive care unit (CG: 10.69 ± 9.01 versus GG: 10.05 ± 3.8, *p* = 0.783) or length hospital stay (CG: 4.19 ± 4.07 versus GG: 3.42 ± 1.9, *p* = 0.471) in the two groups (Table 3).

The number of patients who died at 180 days of follow-up in CG was higher than in GG (2 ± 16 versus 0 ± 19, *p* =0.171) but with no statistically significant difference. In the same day, the number of patients attending to the global mortality in CG was higher than in GG (5 ± 16 versus 4 ± 19, *p* = 0.381) but no statistically significant difference (Table 3).

### 3.4. Hemodynamic Changes in the GDHT Group

**Figure 3:**
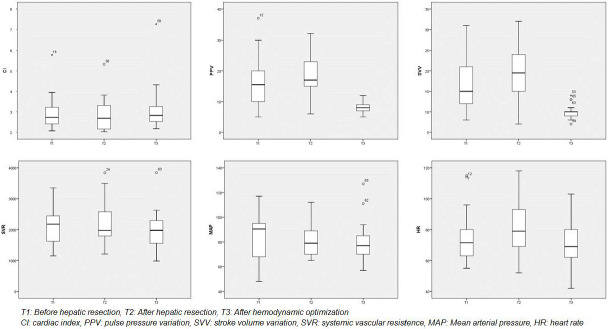
Hemodynamic changes in the GDHT group.

After hepatic resection and hemodynamic optimization according to protocol (Figure 1), there were statistically significant changes in VPP (T2: 19.37 ± 7.96 versus T3: 8.47 ± 1.5, *p* = 0.001), VVS (T2: 19.84 ± 7.05 versus T3: 9.95 ± 1.31, *p* = 0.001) and HR (82.26 ± 18.19 versus 20.53 ± 14.02, *p* = 0.001). In all other variables (CI, SVR, MAP) and times (T2-T1 and T3-T2) there are no statistically significant changes (Table 4 and Figure 3).

## 4. Discussion

Haemodynamic optimization protocols aim to optimize preload and improve cardiac output and tissue perfusion [17]. Personalized haemodynamic optimization includes goal-directed haemodynamic therapy (GDHT). The main aim is to administer the fluid that benefits the patient by avoiding fluid overload, since this results to worse clinical outcomes and increase morbidity and mortality [18,19].

The data from this study is to demonstrate how GDHT is able to administer less fluid to the patient during the dynamic phase after hepatic resection. In addition, the proposed restrictive therapy versus liberal therapy guided by CVP allows less fluid administration during the static phase leading to less surgical time and less bleeding during liver resection. It is therefore one of the first randomized studies in hepatic resection that show how GDHT leads to decreased fluid administration and less intraoperative blood transfusion by using the variables SVV and PPV derived from the radial artery pulse contour.

Other clinical trials of GDHT had also used, as in our study, colloid solutions for intraoperative medical management and ensure an optimized cardiac output, without renal failure [20]. Intraoperative diuresis was significantly lower in the GDHT group, although no increased rate of renal dysfunction was found in the postoperative period. We did not find differences in creatinine and urea parameters (Table 2) in the postoperative period.

In our study, the GDHT group received a restrictive therapy until the end of the hepatic resection which leaded to increased values of the variables PPV, SVV, and CI that showed the expected good response to volume administration. After the administration of the colloid solution, these values were normalized and the heart rate decreased. Therefore, these dynamic parameters would be a better predictor of volume administration than the classic CVP and PAOP that could be altered by the manipulation of the abdominal cavity, the use of vasoconstrictors, mechanical ventilation, etc., and do not correlate with changes in CI after volume expansion [21]. In this way, there are studies that have reported that the CVP can be used as a guide to fluids in hepatic surgery [22], but as it is stated in our study, the patients assigned to the control group received much more volume and blood transfusion.

Several authors have reported that indicate that slight pulmonary edema may occur after liver resection, so patients may benefit from the fluid restriction therapy that we performed in the GDHT group [23]. Some authors have indicated that measuring CVP is not essential to improve hemodynamic control during liver resection [24].

Detailed analysis of intraoperative parameters suggests that, in the GDHT group, volume expansion was required following hepatic resection to achieve optimal values for SVV, PPV, CI, and MAP. We propose that the structured algorithm used in this study facilitated timely fluid administration without hesitation due to concerns about volume overload [25]. The intervention—modification of hemodynamic therapy—was initiated intraoperatively, with the rationale of implementing early goal-directed therapy to maximize its benefits throughout the perioperative period.

Our findings revealed a significant reduction in systemic vascular resistance (SVR) and a concomitant increase in cardiac index (CI) after hepatectomy. These hemodynamic changes are consistent with those reported by Bharathy et al. [26], and may be attributed to the release of splanchnic mediators such as nitric oxide, endotoxins, and pro-inflammatory cytokines [27].

Pearse et al. [28] demonstrated a reduction in postoperative complications following GDHT, attributed to enhanced global oxygen delivery via volume optimization and inotropic support. Their hypothesis was that improved tissue oxygenation promotes healing and reduces infection rates. Although we did not directly measure tissue oxygen demand or partial pressure of oxygen, our algorithm aimed to optimize intravascular volume and CI, likely contributing to improved tissue perfusion and oxygenation.

In our study, the GDHT group exhibited fewer postoperative complications, shorter hospital stays, and lower mortality rates, although these differences did not reach statistical significance. Larger studies in different surgical populations have demonstrated significant benefits of GDHT [29].

Importantly, the monitoring system used in our study was minimally invasive, requiring only a single arterial line. This feature enhances its applicability across a wide range of surgical procedures [23].

Our study had some limitations such as the reduced number of patients, since it is a study of major hepatic surgery and therefore we could not establish a statistically significant difference in the primary endpoint. The anesthesiologists, surgeons and nurses could not be blinded due to the need to use the proAQT device. Furthermore, the parameters of SVV, PPV and CI in the control group were not monitored in order not to interfere with hemodynamic management, although such practice would have been of interest later on compared to the GDHT group. The proposed algorithm could not be established as superior to others used previously and new trials would be needed in the future [30]. Both groups were treated by anesthesiologists with extensive clinical experience. During the static phase in the control group, a liberal fluid administration strategy was employed, reflecting the standard perioperative practice at our institution at the time of the study. Although Eadyn was monitored intraoperatively, it was not systematically recorded for all patients and therefore could not be included in the final analysis.

The results of this study would not be transferable to all patients undergoing liver resection since pulse wave analysis only works reliably in patients without severe arrhythmias and controlled mechanical ventilation.

Finally, the limited sample size and the single-center design restrict the generalizability of our findings. Therefore, this study should be considered exploratory, and we hope it will serve as a foundation for future multicenter trials with greater statistical power.

Personalized, goal-directed therapy optimizes intraoperative fluid administration during major liver resection and reduces surgical times and blood transfusions. We observed fewer complications, hospital stays and mortality at 180 days, but without statistical significance.

Continuous arterial pressure monitoring during major hepatic resection facilitates the use of pulse contour analysis systems, thereby enabling a more rational and individualized approach to perioperative fluid management in patients undergoing hepatectomy. Further prospective studies are warranted to evaluate the impact of personalized goal-directed therapy on perioperative outcomes in this population. Whenever feasible, the implementation of minimally invasive monitoring technologies should be prioritized, as they may contribute to improved quality of care and reductions in postoperative morbidity and mortality.

## Figures and Tables

**Table 1:** Baseline laboratory parameters and demographic subject characteristics.

Demographic Characteristics and Baseline Laboratory Parameters	Control N = 16	GDHT N = 19	*p*-Value
Gender (male)	10 ± 6	11 ± 8	0.528
Age (years)	68.38 ± 8.9	62.37 ± 12.3	0.116
Weight (kg)	76.44 ± 14.4	76.47 ± 15.1	0.99
ASA (II/III)	10 ± 6	13 ± 6	0.495
Cardiovascular disease	5 ± 16	2 ± 19	0.135
Respiratory disese	2 ± 16	3 ± 19	0.598
Preoperative Hemoglobin (mg/dL)	13.3 ± 1.5	13.6 ± 1.4	0.541
Prothrombine Activity (%)	97.31 ± 5.3	97 ± 5.5	0.868
Platelet count (×10^3^/mm^3^ )	23 ± 9.4	21 ± 6.1	0.456
Bilirrubin (mg/dL)	1.2 ± 1.6	0.88 ± 0.6	0.416
Glucose (mg/dL)	108.7 ± 33.3	119.7 ± 41.4	0.399
Creatinin (mg/dL)	0.9 ± 0.21	0.8 ± 0.26	0.235
Amylase (UI/L)	47.07 ± 24.04	35.33 ± 14.4	0.133
AST/GOT (UI/L)	37.55 ± 39.5	41.47 ± 40.02	0.806
ALT/GPT (UI/L)	44.1 ± 60.8	45.8 ± 72.1	0.968

ASA: American Society of Anesthesiology. AST: Aspartate transaminase. ALT: Alanine transaminase. GOT: Glutamic Oxaloacetic Transaminase, GPT: Glutamate Pyruvate Transaminase. GDHT: Goal-directed hemodynamic therapy. Cardiovascular Disease: (hypetensión, coronary artery disesase, heart block, etc.).

**Table 2:** Intraoperative laboratory parameters.

Laboratory Parameters Before Resection Hepatic	Control N = 16	GDHT N = 19	*p*-Value
Hemoglobin (mg/dL)	12.2 ± 1.7	12.13 ± 1.6	0.821
Hemtocrit (%)	26.6 ± 5.2	36.6 ± 4.9	0.984
Prothrombine Activity (%)	86.88 ± 11.1	93.26 ± 6.07	0.045
AST/GOT (UI/L)	171.7 ± 136.7	141.21 ± 129.17	0.502
ALT/GPT (UI/L)	169.44 ± 139.6	108.53 ± 92.2	0.132
Glucose (mg/dL)	154.1 ± 29.7	162.21 ± 55.02	0.229
Creatinin (mg/dL)	0.9 ± 0.211	0.8 ± 0.26	0.229
Lactate (mg/dL)	14.8 ± 9.7	12.4 ± 7.9	0.429
pH	7.38 ± 0.05	7.3 ± 0.05	0.968
Base excess	−1.5 ± 3.3	−1.5 ± 2.8	0.946
**Laboratoy Parameters After Resection Hepatic**	**Control** **N = 16**	**GDHT** **N = 19**	***p*** **-Value**
Hemoglobin (mg/dL)	11.7 ± 1.8	11.2 ± 1.9	0.458
Hemtocrit (%)	35.3 ± 5.7	35.9 ± 5.6	0.458
Prothrombine Activity (%)	79.06 ± 16.69	83.21 ± 11.4	0.392
AST/GOT (UI/L)	537.06 ± 362.19	313.5 ± 214.2	0.03
ALT/GPT (UI/L)	407.88 ± 246.41	236.89 ± 160.4	0.019
Glucose (mg/dL)	173.25 ± 35.7	146.84 ± 36.1	0.038
Creatinin (mg/dL)	1.01 ± 0.35	0.89 ± 0.2	0.278
Lactate (mg/dL)	23.5 ± 14.06	18 ± 13.06	0.239
pH	7.31 ± 0.06	7.32 ± 0.05	0.495
Base excess	−5.1 ± 4.66	−2.7 ± 2.8	0.278
**Laboratoy Parameters 3rd Postoperative Day**	**Control** **N = 16**	**GDHT** **N = 19**	***p*** **-Value**
Hemoglobin (mg/dL)	10.32 ± 1.81	10.21 ± 1.7	0.849
Hemtocrit (%)	30.93 ± 5.49	20.98 ± 5.35	0.978
Prothrombine Activity (%)	72.31 ± 14.82	73.05 ± 9.79	0.861
AST/GOT (UI/L)	166.38 ± 89.75	174.5 ± 152.29	0.86
ALT/GPT (UI/L)	236.81 ± 138.58	243.32 ± 190.35	0.91
Glucose (mg/dL)	110.75 ± 36.5	115.05 ± 30.6	0.707
Creatinin (mg/dL)	1.29 ± 1.16	1.04 ± 0.826	0.462
Lactate (mg/dL)	10.25 ± 3.55	10.11 ± 4.2	0.415
pH	7.39 ± 0.03	7.39 ± 0.05	0.666
Base excess	1.7 ± 3.4	1.8 ± 2.06	0.928

AST: Aspartate transaminase. ALT: Alanine transaminase. GOT: Glutamic Oxaloacetic Transaminase, GPT: Glutamate Pyruvate Transaminase.

**Table 3:** Fluid management and others outcomes.

Fluid Management	Control N = 16	GDHT N = 19	*p*-Value
Blood loss (mL)	728.13 ± 618.59	292.63 ± 274.06	0.009
Static phase volumen infused (mL)	1403.13 ± 1146.51	276.32 ± 189.56	0.001
Dynamic phase volumen infused (mL)	1450 ± 815.06	849.47 ± 669.74	0.023
Total volumen infused (mL)	2853.13 ± 1432.18	1125.79 ± 751.2	0.001
Urinary output (mL)	430.63 ± 310.26	206.84 ± 133.2	0.007
Others outomes			
Duration of resection (min)	82.19 ± 29.9	49.05 ± 18.5	0.001
Duration of surgery (min)	232.5 ± 65.9	210.42 ± 59.25	0.304
Intraoperative use of vasopressor (n)	6 ± 16	6 ± 19	0.495
Intraoperative transfusion (n)	6 ± 16	0 ± 19	0.005
Postoperative (Intensive Unit) use of vasopressor (n)	6 ± 16	4 ± 19	0.311
Postoperative transfusion (n)	7 ± 15	5 ± 19	0.192
Complications (n)	8 ± 16	7 ± 18	0.38
Hospital length of stay (days)	4.19 ± 4.07	3.42 ± 1.9	0.471
Intensive care unit length of stay (days)	10.69 ± 9.01	10.05 ± 3.8	0.783
Mortality at 180 days follow-up (n)	2 ± 16	0 ± 19	0.171
Global mortality (n)	5 ± 16	4 ± 19	0.381

**Table 4:** Hemodynamic changes in the GDHT group.

	T1	T2	T3	*p*-Value T2 vs. T1	*p*-Value T3 vs. T2
CI (L/min/m^2^)	2.85 ± 0.54	2.7 ± 0.58	3 ± 0.61	0.732	0.087
PPV	17.21 ± 8.18	19.37 ± 7.96	8.47 ± 1.5	0.286	0.001
SVV	16.53 ± 5.81	19.84 ± 7.05	9.95 ± 1.31	0.088	0.001
SVR (dyn·s/cm^5^)	2134.79 ± 584.76	2199.47 ± 549	1980 ± 592.46	1	0.159
MAP (mmHg)	83.58 ± 18.74	80.32 ± 13.42	79.68 ± 16.9	0.494	0.732
SAP (mmHg)	114.74 ± 23.31	112 ± 19.37	117.58 ± 20.88	0.702	0.153
DAP (mmHg)	64 ± 14.58	64.37 ± 13.03	60.88 ± 14.38	0.887	0.26
HR (beats min^−1^)	75.74 ± 16.41	82.26 ± 18.19	70.53 ± 14.02	0.016	0.001

T1: Before hepatic resection, T2: After hepatic resection, T3: After hemodynamic optimization; CI: cardiac index, PPV: pulse pressure variation, SVV: stroke volume variation, SVR: systemic vascular resistance, MAP: Mean arterial pressure, SAP: systolic arterial pressure, DAP: diastolic arterial pressure, HR: heart rate.

## Data Availability

The original contributions presented in this study are included in the article. Further inquiries can be directed to the corresponding authors.

## Abbreviations

The following abbreviations are used in this manuscript:

SVV	Stroke volume variation.
PPV	Pulse pressure variation.
PM	Pringle maneuver.
PVC	Central venous pressure.
PAOP	Pulmonary artery occlusion pressure.
Eadyn	Elastance
GDHT	Goal-directed hemodynamic therapy.
CO	Cardiac output.
IC	Cardiac index.
PACU	Post-anesthetic care unit.
ASA	American Society of Anesthesiology.
AST	Aspartate transaminase.
ALT	Alanine transaminase.
SVR	Systemic vascular resistance.
MAP	Mean arterial pressure.
SAP	Systolic arterial pressure
DAP	Diastolic arterial pressure
HR	Heart rate.

## References

[1] Abdelmalak J., Strasser S.I., Ngu N., Dennis C., Sinclair M., Majumdar A., Collins K., Bateman K., Dev A., Abasszade J.H. (2023). Improved Survival Outcomes with Surgical Resection Compared to Ablative Therapy in Early-Stage HCC: A Large, Real-World, Propensity-Matched, Multi-Centre, Australian Cohort Study. Cancers.

[2] Ferrero A., Russolillo N., Viganò L., Lo Tesoriere R., Muratore A., Capussotti L. (2010). Does Pringle maneuver affect survival in patients with colorectal liver metastases?. World J. Surg..

[3] Liu T.S., Shen Q.H., Zhou X.Y., Shen X., Lai L., Hou X.M., Liu K. (2021). Application of controlled low central venous pressure during hepatectomy: A systematic review and meta-analysis. J. Clin. Anesth.

[4] Messina A., Grieco D.L., Alicino V., Cecconi M., Teboul J.L., Monnet X. (2025). Assessing fluid responsiveness by using functional hemodynamic tests in critically ill patients: A narrative review and a profile-based clinical guide. J. Clin. Monit. Comput..

[5] Kim J.H. (2017). Should low central venous pressure be maintained during liver transplantation?. Open Anesthesiol. J..

[6] Niemann C.U., Feiner J., Behrends M., Eilers H., Ascher N.L., Roberts J.P. (2007). Central venous pressure monitoring during living right donor hepatectomy. Liver Transplant..

[7] Vos J.J., Kalmar A.F., Struys M.M.R.F., Wietasch J.K.G., Hendriks H.G.D., Scheeren T.W.L. (2013). Comparison of arterial pressure and plethysmographic waveform-based dynamic preload variables in assessing fluid responsiveness and dynamic arterial tone in patients undergoing major hepatic resection. Br. J. Anaesth..

[8] Monnet X., Shi R., Teboul J.L. (2022). Prediction of fluid responsiveness. What’s new?. Ann. Intensive Care.

[9] Pinsky M.R. (2012). Heart lung interactions during mechanical ventilation. Curr. Opin. Crit. Care.

[10] Lorente J.V., Hahn R.G., Jover J.L., Del Cojo E., Hervías M., Jiménez I., Uña R., Clau-Terré F., Monge M.I., Llau J.V. (2023). Role of crystalloids in the perioperative setting: From basics to clinical applications and enhanced recovery protocols. J. Clin. Med..

[11] Messina A., Robba C., Calabrò L., Zambelli D., Iannuzzi F., Molinari E., Scarano S., Battaglini D., Baggiani M., De Mattei G. (2021). Association between perioperative fluid administration and postoperative outcomes: A 20-year systematic review and meta-analysis of randomized goal-directed trials in major visceral/noncardiac surgery. Crit. Care.

[12] Calvo-Vecino J.M., Ripollés-Melchor J., Mythen M.G., Casans-Francés R., Balik A., Artacho J.P., Martínez-Hurtado E., Romero A.S., Pérez C.F., de Lis S.A. (2018). Effect of goal-directed haemodynamic therapy on postoperative complications in low–moderate risk surgical patients: A multicentre randomised controlled trial (FEDORA trial). Br. J. Anaesth..

[13] Cecconi M., Fasano N., Langiano N., Divella M., Costa M.G., Rhodes A., Rocca G.D. (2011). Goal-directed haemodynamic therapy during elective total hip arthroplasty under regional anaesthesia. Crit. Care.

[14] Kukralova L., Dostalova V., Cihlo M., Kraus J., Dostal P. (2022). The impact of individualized hemodynamic management on intraoperative fluid balance and hemodynamic interventions during spine surgery in the prone position: A prospective randomized trial. Medicina.

[15] Hopewell S., Chan A.W., Collins G.S., Moher D., Schulz K.F., Altman D.G. (2025). CONSORT 2025 statement: Updated guideline for reporting randomised trials. Can. J. Emerg. Med..

[16] Keats A.S. (1978). The ASA classification of physical status—A recapitulation. Anesthesiology.

[17] Li X., Zhang Q., Zhu Y., Yang Y., Xu W., Zhao Y., Liu Y., Xue W., Fang Y., Huang J. (2023). Effect of perioperative goal-directed fluid therapy on postoperative complications after thoracic surgery with one-lung ventilation: A systematic review and meta-analysis. World J. Surg. Oncol..

[18] Pearse R.M., Harrison D.A., MacDonald N., Gillies M.A., Welch N.J., Griggs K., Scott M., Bellingan G., Hopkins P., Rowan K.M. (2014). Effect of a perioperative, cardiac output-guided hemodynamic therapy algorithm on outcomes following major gastrointestinal surgery: A randomized clinical trial and systematic review. JAMA.

[19] Joosten A., Wilets I., Naik B.I., Jabaudon M., Lehot J.J., Cannesson M. (2019). Long-term impact of crystalloid versus colloid solutions on renal function and disability-free survival after major abdominal surgery. Anesthesiology.

[20] Rahimi P., Aşar S., Soylu N.B., Yücel Yenice T., Canan E., Çukurova Z. (2025). Driving down mortality: A 12-year retrospective cohort analysis of mechanical power and driving pressure in ventilated ICU patients. Medicina.

[21] Suarez D. (2022). Changes in arterial pressure during mechanical ventilation. J. Labor Childbirth.

[22] Hofer C.K., Zalunardo M.P., Klaghofer R., Spahn D.R. (2004). Therapeutic impact of intra-operative transoesophageal echocardiography during noncardiac surgery. Anaesthesia.

[23] Chaves R.C.F., Barbas C.S.V., Queiroz V.N.F., Neto A.S., Deliberato R.O., Pereira A.J., Timenetsky K.T., Silva Júnior J.M., Takaoka F., de Backer D. (2024). Assessment of fluid responsiveness using pulse pressure variation, stroke volume variation, plethysmographic variability index, central venous pressure, and inferior vena cava variation in patients undergoing mechanical ventilation: A systematic review and meta-analysis. Crit. Care.

[24] Tan S.Y.L., Hwang N.C. (2022). Total intravenous anesthesia for liver resections: Anesthetic implications and safety. Korean J. Anesthesiol..

[25] Yerdon A., Taylor K., Woodfin K., Richey R., McMullan S., Chappell D. (2025). Goal-directed therapy: What is the goal again?. Perioper. Med..

[26] Bharathy K.G.S., Shenvi S. (2023). Portal hemodynamics after living-donor liver transplantation: Management for optimal graft and patient outcomes—A narrative review. Transplantology.

[27] Sparrelid E., Olthof P.B., Dasari B.V.M., Erdmann J.I., Santol J., Starlinger P., Gilg S. (2022). Current evidence on posthepatectomy liver failure: Comprehensive review. BJS Open.

[28] Pearse R., Dawson D., Fawcett J., Rhodes A., Grounds R.M., Bennett E.D. (2005). Early goal-directed therapy after major surgery reduces complications and duration of hospital stay. A randomised, controlled trial. Crit. Care.

[29] Kuper M., Gold S.J., Callow C., Quraishi T., King S., Mulreany A., Bianchi M., Conway D.H. (2011). Intraoperative fluid management guided by oesophageal Doppler monitoring. BMJ.

[30] Yoshino O., Perini M.V., Christophi C., Weinberg L. (2017). Perioperative fluid management in major hepatic resection: An integrative review. Hepatobiliary Pancreat. Dis. Int..

